# Integrated exposure assessment of sewage workers to genotoxicants: an urinary biomarker approach and oxidative stress evaluation

**DOI:** 10.1186/1476-069X-10-23

**Published:** 2011-03-24

**Authors:** Hamzeh Al Zabadi, Luc Ferrari, Irène Sari-Minodier, Marie-Aude Kerautret, Aziz Tiberguent, Christophe Paris, Denis Zmirou-Navier

**Affiliations:** 1 An-Najah National University, School of Medicine-Public Health Department, Nablus, Palestine; 2 INSERM U954, School of Medicine, 54505 Vandœuvre-lès-Nancy, France; 3 Nancy University Faculty of Pharmacy, 54000 Nancy, France; 4 Nancy University School of Medicine, 54505 Vandœuvre-lès-Nancy, France; 5 Aix-Marseille University School of Medicine, EA 1784, 13005 Marseille, France; 6 Paris City Hygiene laboratory, Paris, France; 7 Occupational medicine service of the City of Paris, France; 8 EHESP School of Public Health-IRSET, 35000 Rennes, France

## Abstract

**Background:**

Sewage workers are exposed to multiple chemicals among which many are suspected genotoxicants. Therefore, they might incur DNA damage and oxidative stress. We aimed to explore integrated urinary biomarkers, assessing the overall urine genotoxicity by *in vitro *comet and micronucleus assays and measuring urinary 8-oxo-2'-deoxyguanosine.

**Methods:**

During three consecutive working days, polycyclic aromatic hydrocarbons and volatile organic compounds were sampled in workplace air of 34 sewage and 30 office workers, as indicators of airborne exposure. The last day, subjects collected their 24 hours urine. Genotoxicity of urinary extracts was assessed by comet and micronucleus assays on a HepG2 cell line. Using competitive enzymatic immunoassay we evaluated the 24 hours urinary 8-oxo-2'-deoxyguanosine excretion. Benzo(a)pyrene toxicity equivalent factors and inhalation unit risk for Benzo(a)pyrene and benzene were used to give an estimate of cancer risk levels.

**Results:**

Workplace air concentrations of polycyclic aromatic hydrocarbons (e.g. 23.7 [range 2.4-104.6] ng.m^-3 ^for fluoranthene) and volatile organic compounds (e.g. 19.1 ± 2.9 [standard error] μ.m^-3 ^for benzene) were elevated in sewage compared to office workplaces (*P *< 0.01) and corresponded to an increased lifetime cancer risk. The urinary extracts of sewage workers showed higher genotoxicity (*P *< 0.001) than office workers.

**Conclusions:**

The integrated and non-specific urinary biomarkers of exposure showed that sewage workers experience exposure to mixtures of genotoxicants in the workplace.

## Background

Sewage workers provide an essential service that contributes to the protection of public health. Their role is to maintain the sewage system through which wastewaters and hazardous agents produced by our urbanized society are disposed of. Sewage system receives deposits of polycyclic aromatic hydrocarbons (PAHs) and solubilised volatile organic compounds (VOCs) from different sources such as traffic exhausts, industries, waste incinerators, and domestic heating via both atmospheric deposition and activities of the society [1,2]. Many other chemicals may also be found in the sewage workplace environment, such as fluorinated hydrocarbons, heavy metals, pesticides, dyes, nitrosamines and polychlorinated biphenyls [3-5]. As a result, sewage workers experience exposure to a wide and complex variety of chemicals many being known or suspected genotoxicants and/or carcinogens [6,7]. Indeed, although scant and not completely consistent, some studies suggest an increased risk of cancer and total mortality [3,8,9] among sewage workers.

This complex exposure varies along time, locations, concentration levels and pathways. It is often intermittent, occasionally acute, over a chronic background. These exposure fluctuations cannot be easily captured by measuring a single or a limited number of pollutants at a given time and place or by exploring only one route of exposure [10]. An attractive alternative approach is the use of biomarkers. This may be achieved by collecting samples from easily obtainable biological material in order to assess the total individual exposure to non-specific substances with which subjects come in contact through different routes (lung, skin and gastrointestinal tract) and sources (air, diet, lifestyle or occupation) [11]. In addition, the use of non-specific biomarkers of exposure and of early effects in exposed workers, together with careful assessment of workplace at various locations and over time, could be a tool to gain insight into the hazardous potency of such complex occupational settings. It might also support the link between occupational exposure and the risk of adverse health effects [10].

In the human body, toxicants like PAHs and VOCs may appear as intact compounds or as metabolites, in particular in the urine, within a few hours or days following exposure [12]. Therefore, urine offers the advantage to represent the effective overall body uptake through different routes of exposure, to account for personal metabolism activities and to be a non-invasive medium. However, specific biomarkers fall short to express a complex exposure to a variety of compounds, a situation that sewage workers experience, among other occupations. Many compounds encountered in the sewage system are genotoxicants [3]. Urine genotoxicity assessment might thus be an appropriate approach to integrate the exposure to an array of genotoxic compounds that eventually result in a variety of urinary excreted metabolites which are too many to be individually quantified. Hence, the genotoxic potency of urine might be used as a biomarker of exposure to genotoxicants.

When the genotoxicants are incorporated into the human body, their metabolism may generate reactive oxygen species. The latter might interact with cell nucleus DNA, leading to oxidative DNA damage [13]. The 8-oxo-2'-deoxyguanosine (8-oxodG) is a biomarker of guanine oxidation in DNA and one of the most easily-formed DNA lesions. The DNA base excision repair pathway of oxidant induced bases recognizes 8-oxodG as a threatening lesion; this results in its excretion in human urine without further metabolism [14]. Urinary assessment of this biomarker is increasingly used as a non-invasive biomonitoring approach that estimates the overall DNA oxidative stress produced in the body, while blood assessment of this biomarker represents only part of this oxidative stress [13]. The DNA damage represented by 8-oxodG is important in the pathogenesis of many diseases, including cancer [14].

There is no information on the levels of DNA oxidized bases, mainly 8-oxodG, among underground sewage workers. However, male workers exposed to fly ash at solid waste incinerators, showed a significant increase in the mean levels of urinary 8-oxodG with duration and level of exposure [15]. Data on personal exposure to PAHs and VOCs in the workplace air of underground sewage workers are not available. However, many studies have found these chemicals in wastewater treatment plants [16-18], in the air of municipal solid waste [19] and in sewage sludge [20].

As part of a biomarker study to assess exposure of sewage workers to complex chemical mixtures [21], the aims of the present study were: (1) to evaluate the overall genotoxicity of urinary extracts of Parisian underground sewage workers, as urinary biomarkers of exposure, and compare it with urines from office workers by comet and micronucleus assays, (2) to explore early effects through the assessment of DNA oxidative stress measured as the urinary excretion of 8-oxodG. In addition, we compared workplace air concentrations of PAHs and VOCs, used as indicators of airborne exposure in these two occupations.

## Methods

### Study population, setting and design

The study protocol has been described in detail elsewhere [21]. Briefly, 34 underground sewage workers and a control group of 30 office workers from the city of Paris were recruited on a weekly basis and over a 10 months period (July 2008-April 2009). All were male volunteers, current non-smokers since at least six months, aged 20-60 years, employed at the same function for at least six months with no history of chronic or recent illness. Interviews and biological sampling were conducted in the framework of regular occupational medical visits at the offices of occupational and preventive medicine of Paris municipality. The study was approved by the local ethical committees and has been conducted according to the Helsinki Declaration. A signed informed consent was obtained from each participant.

During three consecutive days of work shifts prior to the medical visit, workplaces indoor air concentrations for 13 PAHs and 12 VOCs were measured. Each subject collected 24 hours urine in a sterile plastic bottle in the last day of air sampling. During the medical visit that took place on Thursdays or Fridays, subjects filled in two self-administered questionnaires. One for socio-demographic factors, non occupational exposures (especially possible PAHs and VOCs exposures related to commuting, to area of residence and indoor sources), medical history, lifestyle (smoking history, including passive smoking, alcohol consumption and medications), usage of protection equipments and other putative confounders. The second for diet habits including barbecue usage and daily intake of fruits and vegetables.

### Chemicals, media and reagents

Unless otherwise specified, all chemicals and culture media used were purchased from Sigma-Aldrich Chimie S.A.R.L (L'Isle d'Abeau Chesnes, France). For 8-oxodG, the 96-well kits were purchased from CliniSciences SA, Montrouge, France (origin: StressMarq Biosciences Inc., Victoria, BC Canada) [22].

### Workplaces air sampling and analysis

The sampling equipments were placed in a backpack and handed to one subject per work team (a team being generally composed of two to three subjects). The backpack contained a personal active sampling ChemPass pump (Rupprecht and Patastnick Co., Inc. NY, USA) with a calibrated flow rate of 4 L/minute, to measure PAHs. It was carried during the sewage activities or placed on a desk nearby the office workers. A VOCs passive sampling badge (Radiello code 145, Sigma-Aldrich, France) was also provided and attached to workers' clothes near the breathing zone.

PAHs in particulates and gaseous forms were collected on a quartz filter (Supelco 21038, 32 mm of diameter) and a cartridge containing polyurethane foam (Supelco ORBO 2-0600); respectively. The pump was equipped with a timer and subjects had to turn it on during work shifts and off when finished. The flow rate was measured before and after sampling and the sample was rejected if the difference was greater than 10%. For analysis, the filter and the foam were subjected to extraction by accelerated solvent (hexane-acetone; 50/50, v/v). The extract was concentrated in a water bath at 35°C using automatic evaporator (Turbo Vap II Zymark), then under nitrogen flow evaporator (N-Evap, MA, USA) until obtaining an oily drop. The drop was taken by 1 ml of acetonitrile solvent compatible high performance liquid chromatography by which the extracts were analyzed using a fluorescence emission detector (Waters 2475). The quantification analysis was carried out according to the calibrated response of standard solutions of marked-mixture for the selected PAHs. VOCs were analysed by coupling gas chromatography and mass spectrometry, as described previously [21].

The workplace collection of the PAHs and VOCs were performed from Monday to Thursday for sewage workers and from Monday to Wednesday for office workers. For comparison with ambient air pollution, results from the Paris air quality monitoring network (AIRPARIF) were retrieved. The average concentrations at the same or at the nearest days of the study period, were obtained from the closest monitoring stations relative to the sampled underground workplaces, with each time two types of monitors: one measuring background air quality and the other close to traffic sources.

Thirteen PAHs were quantified [twelve listed as priority pollutants by the United States Environmental Protection Agency (U.S. EPA)] plus benzo(j)fluoranthene. Of these, four are in gaseous form (phenanthrene, anthracene, fluoranthene and pyrene) and the rest are particles. Twelve VOCs were also quantified.

### Urinary fraction extraction

Extraction of the urinary organic fraction was performed on a Sep Pak Vac C18 cartridge (Waters, Saint-Quentin en Yvelines) [21]. Briefly, the volume of the 24 hours urine was measured and a sample of 150 ml was immediately coded and frozen at -20 C° for each subject. For the assays, samples were then thawed and 50 ml were centrifuged at 3000 t/minute for 5 minutes. The supernatant (40 ml) was collected in a sterile tube. The organic fraction was extracted on a Column Sep Pak^® ^Vac C18 Cartridges on an aspirated tray (J.T. Baker spe -12G). The cartridge was washed 2 × 3 ml of absolute methanol, then 3 × 3 ml of ultra-pure water. It was then loaded with the 40 ml urine. The column was washed 2 × 3 ml of ultra-pure water and the adsorbed organics were eluted with 3 × 3 ml of absolute acetone (Carlo, CAS 67-64-1). The eluate was evaporated at 45°C under nitrogen stream (Tech Lab Faster-Chemfree, France) until complete dryness. The residue was suspended in 500 μl DMSO and stored at -20°C. All operations were done at room temperature.

### Cell culture and exposure

HepG2 cells (ATCC, catalog number HB-8065) were routinely cultured in the laboratory in monolayers. They were maintained in EMEM (Eagle's minimum essential medium, 9.6 g/L) supplemented with 10% FBS (Fetal bovine serum) and 1% antibiotics solution (penicillin 10000 U/ml; streptomycin 10 mg/ml), sodium carbonate (2.2 g/L), Hepes (5.96 g/L), and sodium pyruvate (0.11 g/L). The pH of cultured mediums was maintained between (7.2-7.4). Cells were grown at 37 C° in a humidified 5% CO2 and 95% air atmosphere. HepG2 cells were cultivated for 24 hours in 5 ml complete culture medium supplemented with 50 μl of organic urine extract. After harvesting and centrifugation, the cell pellet was diluted by the culture medium to 5 × 10^5 ^cell/ml. Trypan blue exclusion test was used to assess toxicity, and cell viability was always ≥ 95%.

### Comet and cytokinesis block micronucleus assays

Comet assay was performed essentially as in Singh et al. [23], with some modifications as in Muller-Pillet et al. [24]. Briefly, after layering the cells on conventional microscopic slides previously sprayed by normal melting point agarose, they were lysed at 4°C in the dark for at least 1 hour (2.5 M NaCL, 100 mM Na2EDTA, 10 mM Tris and 1% Triton X-100, Prolabo 28.817.295 and 10% DMSO, pH = 10). The electrophoresis was conducted (20 V, 0.62 V/cm, 300 mA, 20 minutes) in an electrophoresis gel system (EC340, Maxicell^® ^Primo, Holbrook, New York) filled with electrophoresis buffer (300 mM NaOH, 1 mM Na2EDTA, pH 13). B(a)P 40 μM and DMSO 1% final concentrations (positive and negative controls; respectively) were added with each electrophoretic run. After neutralizing the slides in 0.4 M Tris buffer, they were stained with 40 μl ethidium bromide (2 μg/ml in ultra-pure water). 50 cells (two slides/subject and 25 cells/slide) were examined for DNA migration using an Olympus BX40 fluorescence microscope (Olympus, Tokyo, Japan). We quantified DNA damage by a computerized image analysis system (Komet 4.02 software, Kinetic Imaging, UK) in evaluating the percent DNA tail parameter. The gel was scanned in a systematic way and the comets represented the whole gel. Edges and areas around air bubbles were avoided. Clouds images (indicative of dead cells) were also excluded from acquisition and analysis.

For micronucleus assay, we adapted the assay described by Fenech [25]. Briefly, at time = 0, HepG2 cells were grown in 10 ml complete medium for 24 hours. Then, in a freshly 10 ml diluted medium supplemented with 100 μl of organic urine extract (time = 24 hours). Subsequently, at time = 44 hour, the cells were cultivated in a fresh complete culture medium supplemented with 3 μg/ml cytochalasin-B final concentration. At time = 72 hour, cells were rinsed in cold hypotonic KCL solution (0.075 M, Prolabo 26.764.298) and then fixed in CARNOY solution (methanol, Carlo Erba 525.102: acetic acid, Prolabo 20.104.298, 3:1 v:v). The air-dried slides were stained with 40 μg/ml Acridine orange solution in dark. The slides were examined at 200-fold magnification by an Olympus BX fluorescence microscope (Olympus, Tokyo, Japan). We evaluated the frequency of the micronuclei (MNi) formation in 1000 binucleated cells (BNed)/slide/subject. We respected Fenech [25] criteria in scoring the MNi. Proliferation and cytotoxicity were assessed by calculating the nuclear division index (NDI) on 150 viable cells according to Fenech [25] formula. NDI mean difference between exposed and non-exposed and between positive and negative controls (B(a)P 40 μM and DMSO 1% final concentrations; respectively) was always acceptable and below 25% [26].

### Analysis of 24 hours urinary 8-oxodG

The urine aliquots (1 ml each) were kept at -20°C. They were thawed at room temperature immediately before analysis. 8-oxodG was measured with a competitive enzymatic immunoassay (EIA) kit. This assay utilizes a specific 8-oxodG monoclonal anti-body (Catalog# SKC-120A), 8-oxodG-acetylcholinesterase (AChE) conjugate and an anti-mouse IgG-coated plate. The manufacturer provided the protocol of analysis [22]. To insure the accuracy and reproducibility of results, each sample was assayed at two dilutions (1/300 and 1/400) and each at duplicates. The 24 hours 8-oxodG excretion was expressed relative to body weight (pmole/kg/24 h) [27,28]. Creatinine in 24 hours urine was determined photometrically as picrate, according to Jaffé method [29].

### Statistical analysis

PAHs concentrations are presented as mean (range) while VOCs are exhibited as mean ± SE. For analyses of associations between biomarkers and exposure, the workplace air concentrations (26 measurements) were assigned to each subject within a team as his exposure level. Chi square and Fisher exact tests were used to analyse the differences between categorical variables. ANOVA was used to compare the mean differences of quantitative variables, and to evaluate the effect of some socio-demographic and putative confounding factors on comet and micronucleus results. Multiple linear regression was conducted to evaluate the level of 8-oxodG among the two study groups while adjusting for possible confounding factors. Multiple linear regression were also implemented to assess the association between exposure to occupational agents and the two genotoxicity assays while adjusting for confounding variables. Only pollutants significantly associated (P < 0.05) in univariate analysis were tested, while confounders were retained if P < 0.1 in univariate analyses. Effect modification was tested, in particular by age whose distribution was partitioned by the median value into two categories (≤ 39 and < 39 years). SPSS 16 software was used for analysis [30].

To assess and characterize the carcinogenic potency of PAH mixtures, toxicity equivalent factors (TEFs) were used to convert PAH exposure into an estimated B(a)P equivalent. We used the Nisbet and Lagoy [31] TEFs with the exception for benzo(j)fluoranthene where the TEFs proposed by Collins et al. [32] was used. Cancer risk due to inhalation of PAH mixtures was then calculated using the B(a)P inhalation risk cancer unit of 1.1 × 10^-6 ^(ng/m^3^)^-1 ^proposed by the U.S. EPA [33]. We also used the benzene unit risk range of 2.2 × 10^-6 ^to 7.8 × 10^-6 ^(μg/m^3^)^-1 ^[34] to assess the cancer risk by inhalation of benzene.

## Results

### Characteristics of the study population

General characteristics of the study population are summarized in Table 1. The mean ages were 35.9 (standard deviation; SD = 7.5) and 43.3 (SD = 8.2) years in sewage (n = 34) and office (n = 30) workers respectively (P < 0.001). The mean ± SD number of working years in sewage was 7.05 ± 6.9 years. Never smokers were more frequent in sewage than in office workers (P = 0.03). However, sewage workers were more likely to drink alcohol regularly than the control group (P = 0.01), and less likely to eat vegetables than the office workers (P = 0.01). The 24 hours urinary creatinine differed between the two groups (P = 0.003) but was within the normal human male values for both groups. The level of education also distinguished the study groups (P = 0.04). No other difference was seen regarding factors that might influence study-relevant exposures: environmental tobacco smoke (P = 0.87), type of heating system used at home (individual or collective, P = 0.52), declared proximity of homes to industrial installations (P = 0.82) or consumption of barbecue grilled food.

**Table 1:** Population characteristics and exposure factors.

Characteristics and exposure factors	Sewage workers (N = 34)	Office workers (N = 30)	Total Sample (N = 64)	P-value
Age (year)	35.85 ± 7.54	43.30 ± 8.15	39.3 ± 8.6	0.001*

Weight (Kg)	77.8 ± 12.2	76.9 ± 10.6	77.4 ± 11.4	0.75

BMI (Kg/m^2 ^)	25.5 ± 3.3	25.3 ± 4.2	25.4 ± 3.7	0.82

24 hours urinary volume (ml)	1500 ± 691.5	1750 ± 655.8	1617.2 ± 681.4	0.14

24 hours Urinary creatinine (g/l)	1.3 ± 0.55	0.91 ± 0.43	1.2 ± 0.54	0.003*

Place of residence				

-Urban	8 (23.5)	9 (30)	17 (26.6)	0.47

-Suburbs	22 (64.7)	15 (50)	37 (57.8)	

-Rural	4 (11.8)	6 (20)	10 (15.6)	

Marital status				

-Married	17 (50)	10 (33.3)	27 (42.2)	0.20

-Not married	17 (50)	20 (66.6)	37 (57.8)	

Level of education				

-< 12 years of schooling	2 (5.9)	8 (26.7)	10 (15.6)	0.04*

-> 12 years of schooling	32 (94.1)	22 (73.3)	54 (84.4)	

Smoking				

-Never smokers	29 (85.3)	18 (60)	47 (73.4)	0.03*

-Ex-smokers	5 (14.7)	12 (40)	17 (26.6)	

Exposure to passive smoking				

-Yes	20 (58.8)	17 (56.7)	37 (57.8)	0.87

-No	14 (41.2)	13 (43.3)	27 (42.2)	

Alcohol consumption				

-Regularly	23 (67.6)	10 (33.3)	33 (51.6)	0.01*

-Occasionally	11 (32.4)	20 (66.7)	31 (48.4)	

Use of barbecue last week				

-Yes	3 (8.8)	0 (0)	3 (4.7)	0.20

-No	31 (91.2)	30 (100)	61 (95.3)	

Massive physical activity (last two days)				

-Yes	6 (17.6)	4 (13.3)	10 (15.6)	0.74

-No	28 (82.4)	26 (86.7)	54 (84.4)	

Fruits intake				

-Usually	21 (61.8)	23 (76.7)	44 (68.8)	0.29

-Sometimes	13 (26.5)	7 (16.7)	20 (21.9)	

Vegetables intake				

-Usually	19 (55.9)	26 (86.7)	45 (70.3)	0.01*

-Sometimes	15 (44.1)	4 (13.3)	19 (29.7)	

Intake of vitamins/minerals				

-Yes	6 (17.6)	2 (6.7)	8 (12.5)	0.27

-No	28 (82.4)	28 (93.3)	56 (87.5)	

Usage of protective equipments				

-Yes	11 (32)	0 (0)	11 (17)	< 0.001*

-No	23 (68)	30 (100)	53 (83)	

Data are frequencies (percentage) or mean ± SD.

BMI, body mass index (the square of the height over the body weight).

*Statistically significant (P < 0.05).

### Concentrations of PAHs and cancer risk characterization

The mean workplace exposure levels of each PAH compound presented in Table 2 were significantly higher among sewage workers compared to office workers and to ambient air concentrations (23 measurements) (P < 0.01). In general, phenanthrene, fluoranthene and pyrene contribute the largest portion of the total PAHs exposure (e.g. among sewage workers they ranged from 43%, 15%, and 12%; respectively). The other PAHs amount to less than 5%, except anthracene after the traffic monitor ambient air measurements (11.5%). The highest B(a)P concentration was found among sewage workers with a range of 0.5 to 62.1 ng/m^3 ^and a mean value of 7 ng/m^3^.

**Table 2:** The mean (range) (ng.m^-^^3^) concentration at the workplaces and the corresponding nearest ambient air measurements of PAHs, and the derived total [B(a)P]eq and cancer risk estimates.

PAHs	Sewage workplace (n = 26)	Office workplace (n = 26)	Traffic (n = 23)	Urban (n = 23)
Benzo(a)pyrene	6 (0.5-62.1)	0.4 (0.2-2.4)	0.7 (0.1-1.6)	0.5 (0.2-5.8)

Anthracene	6.7 (0.5-32.1)	0.9 (0.1-1.8)	3.2 (0.3-14.5)	0.2 (0.02-0.8)

Benz(a)anthracene	4.6 (0.3-31.8)	0.5 (0.2-2.4)	0.5(0.2-1.2)	0.2 (0.02-0.6)

Benzo(b)fluoranthene	4.3 (0.5-30.1)	0.4 (0.1-2.4)	0.6 (0.2-1.6)	0.3 (0.05-1.1)

Benzo(g,h,i)perylene	4.4 (0.4-24)	0.5 (0.1-3.8)	0.8 (0.2-1.7)	0.3 (0.06-1)

Benzo(k)fluornathene	2.0 (0.2-15.2)	0.2 (0.08-1.2)	0.2 (0.07-0.6)	0.2 (0.01-2.5)

Chrysene	7.7 (1-30)	2.2 (1.1-6.8)	0.7 (0.3-0.2)	0.3 (0.1-0.8)

Dibenz(a,h)anthracene	0.9 (0.01-5.4)	0.1 (0.01-0.5)	0.02 (0.01-0.06)	0.02 (0.01-0.06)

Fluoranthene	23.7 (2.4-104.6)	4.3 (2.5-8.1)	3.8 (2-5)	1.6 (0.8-4.3)

Indeno(1,2,3-cd)pyrene	3 (0.3-15.9)	0.3 (0.08-2.4)	0.4 (0.07-1)	0.2 (0.02-0.7)

Phenanthrene	71.2 (12.5-220)	22.3 (9-43.9)	11.9 (5.2-18.1)	5.2 (2.3-14.2)

Pyrene	19.3 (2.3-78.4)	5.5 (1.2-21.7)	4.8 (2.8-6.3)	1.3 (0.6-3.4)

Benzo(j)fluoranthene	3.9 (0.3-21.6)	0.4 (0.1-3.3)	0.4 (0.03-1.2)	0.2 (0.03-0.8)

				

Total [B(a)P]eq (ng m^-3^)	13.66	1.15	1.08	0.73

B(a)P equivalent lifetime cancer risk	15.02 × 10^-6^	1.26 × 10^-6^	1.19 × 10^-6^	0.80 × 10^-6^

a. B(a)P TEFs according to Nisbet and LaGoy (1992), for benzo(j)fluoranthene Collins et al. (1998) value was applied.

b. The U.S. EPA inhalation unit cancer risk of B(a)P = 1.1 × 10^-6 ^(ng/m^3^)^-1 ^was used (U.S. EPA 2009).

Total [B(a)p]eq, total benzo(a)pyrene equivalent concentration.

Based on these data, single PAHs concentrations were converted into total B(a)P equivalent concentration ([B(a)P]eq) using the TEFs (see statistical analysis) and translated into lifetime cancer risk estimates. The average sewage workers' total [B(a)P]eq exposure value (13.66 ng/m^3^) is more than 10 times greater than those encountered for office workers, traffic and urban ambient air levels (respectively 1.15 ng/m^3^, 1.08 ng/m^3^, and 0.73 ng/m^3^). Table 2 also shows the associated lifetime cancer risks. The PAHs cancer risk level for sewage workers is 1.5 × 10^-5 ^(0.13 × 10^-5 ^for office workers).

### Concentrations of VOCs and related cancer risk characterization

**Figure 1:**
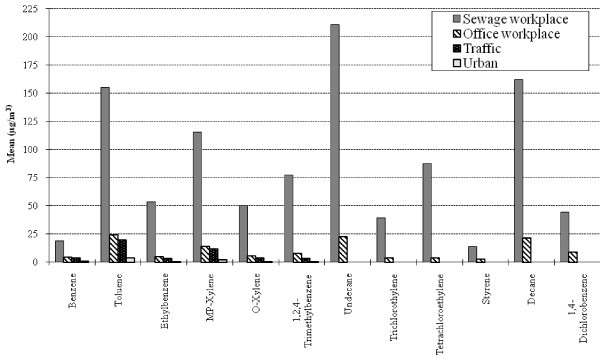
**Volatile organic compounds (VOCs) concentration patterns in the workplaces air and from the nearest outdoor monitoring stations**. Statistically significant higher means concentrations (P < 0.01) among sewage workers compared to all other groups for all substances. Comparison of workplace and ambient air concentrations is incomplete because some VOCs measured in the workplaces are not measured by traffic or background urban monitors (undecane, trichloroethylene, tetrachloroethylene, styrene, decane and 1,4-dichlorobenzene).

Figure 1 presents the mean of each VOC concentrations in the workplace air of sewage and office workers, and in the air of urban and traffic environments nearest to the study locations. A high heterogeneity was observed between the different locations with the highest values found in the sewage workplace (P < 0.01), followed by indoor air of office workers, traffic and urban background ambient air, respectively. Benzene (mean ± SE) concentrations were 19.1 ± 2.9 and 4.1 ± 0.53 μg/m^3 ^among sewage and office workers, respectively; corresponding values were 3.7 ± 0.13 and 1.0 ± 0.09 μg/m^3 ^in traffic and urban background, respectively. Using the Integrated Risk Information System (IRIS) unit risk estimate range [34], the benzene associated lifetime excess cancer risk for sewage workers ranged from 4.2 × 10^-5 ^to 14.9 × 10^-5 ^(0.9 × 10^-5 ^to 3.2 × 10^-5 ^for office workers).

### In vitro genotoxicity assays on urinary extracts

**Figure 2:**
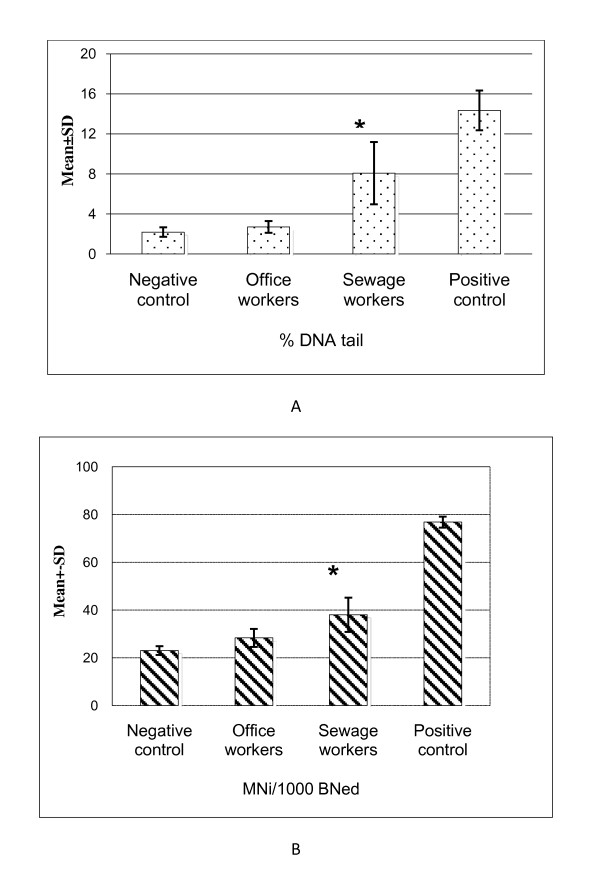
**Effects of 24 h exposure to urine organic extract evaluated by comet and micronucleus assay performed on HepG2 cells**. A) The bars are related to the means ± SD values of percent DNA tail obtained by comet assay. B) The bars are related to means ± SD values of MNi/1000 BNed obtained by Micronucleus assay. For comet assay, nine experiments were performed. For micronucleus assay, five experiments were performed. For both assays and in each experiment, B(a)P 40 μM and DMSO 1% final concentrations were the positive and negative controls; respectively. *Statistically significant (P < 0.001) compared to office workers. BNed, Binucleated cells MNi, micronuclei.

The mean percent DNA tail and MNi/1000 BNed among sewage workers was statistically higher than in office workers [mean ± SD of percent DNA tail = 8.07 ± 3.12 and 2.70 ± 0.58, and mean ± SD of MNi/1000 BNed = 38.02 ± 7.16 and 28.30 ± 3.74, respectively in the two populations [P < 0.001 in both tests] (Figure 2A and 2B).

In multivariate linear regression models, we tested the differences in percent DNA tail and MNi/1000 BNed between the two study occupation groups while adjusting for possible confounders including 24 hours urinary creatinine. The differences between sewage and office worker are, (point estimate and [95% confidence interval]), 5.01 [3.01-7.00] for percent DNA tail and 9.41 [4.47-14.36] for MNi/1000 BNed, respectively. No interaction was observed according to subjects' characteristics.

### In vitro genotoxicity assays and workplace air concentrations

The percent DNA tail was positively associated with age and educational level with a borderline positive effect of alcohol consumption (P = 0.09) and a protective effect of vegetable intake (P = 0.05). MNi/1000 BNed was positively associated with age and alcohol consumption while inversely with vegetable intake. Table 3 also presents the results of the association of percent DNA tail and MNi/1000 BNed with each individual VOC and PAH.

**Table 3:** Univariate analysis for factors associated with genotoxicity assays performed with urine extracts on HepG2 cells: statistical significance of associations (N = 64).

Independent variables	P-value	
	
	Tail DNA percent	MNi/1000 BNed
Socio-demographic characteristics		

Marital status (married/not married)	0.12	0.94

Smoking (ex-smokers/never smokers)	0.16	0.18

Educational level; years (> 12/≤ 12)	0.04	0.23

Age; years (≤ 39/> 39)	0.01	0.02

Alcohol consumption (regularly/occasionally)	0.09	0.05

Vegetable intake (usually/sometimes)	0.05	0.02

VOCs		

Benzene	0.01	0.001

Toluene	0.14	0.004

Ethylbenzene	0.01	0.001

M+P-Xylene	0.002	< 0.001

O-Xylene	0.003	< 0.001

1, 2,4 Trimethylebenzene	0.02	< 0.001

Undecane	0.05	< 0.001

Trichloroethylene	0.07	0.05

Tetrachloroethylene	0.01	0.19

Styrene	0.09	0.002

Decane	0.001	< 0.001

1,4 Dichlorobenzene	0.06	0.02

PAHs		

Benzo(a)pyrene	0.40	0.35

Anthracene	0.02	0.17

Benz(a)anthracene	0.20	0.20

Benzo(b)fluoranthene	0.16	0.22

Benzo(g,h,i)perylene	0.09	0.22

Benzo(k)fluornathene	0.21	0.24

Chrysene	0.01	0.03

Dibenz(a,h)anthracene	0.02	0.51

Fluoranthene	0.04	0.06

Indeno(1,2,3-cd)pyrene	0.08	0.20

Phenanthrene	0.01	0.03

Pyrene	0.03	0.11

Benzo(j)fluoranthene	0.06	0.26

Total [B(a)P]eq	0.15	0.34

Total [B(a)p]eq, total Benzo(a)pyrene equivalent concentration

MNi, micronuclei

BNed; binucleated cells

Results of the multiple linear regression models are presented in Table 4 that exhibits the association between each of the *in vitro *genotoxicity assays and the assigned personal atmospheric exposure variables, controlling for covariates. Effect modification is also accounted for, with age being the sole factor influencing this association. A significantly positive association with the comet test response was seen among older workers only (> 39 years; n = 34) for nine PAHs (the four gaseous PAHs and five out of the nine particulate PAHs). This association with PAHs was not found for the MNi/1000 BNed. All VOCs were significantly associated with MNi/1000 BNed among older workers, while percent DNA tail was only influenced by exposure to benzene, ethylbenzene, m+p-xylene, o-xylene, decane, tri and tetra-chloroethylene. Noteworthy is that no difference in 24 hours urinary volume or creatinine levels between the two age groups was observed (P = 0.91 and 0.20; respectively).

**Table 4:** Association between exposures to workplace toxicants and genotoxicity assays performed on HepG2 cells: multiple linear regression models (N = 64)*.

Independent variable	Tail DNA percent			MNi/1000 BNed		
	
	Reg. coeff #	P-value	95% CI for B	Reg. coeff #	P-value	95% CI for B
VOCs						

Benzene						

≤ 39 yrs	0.03	0.61	-0.08 to 0.14	0.03	0.78	-0.17 to 0.23

> 39 yrs	0.08	0.03	0.01 to 0.16	0.41	0.00	0.23 to 0.56

Ethylbenzene						

≤ 39 yrs	0.02	0.24	-0.01 to 0.05	-0.01	0.80	-0.06 to 0.05

> 39 yrs	0.02	0.05	0.00 to 0.03	0.10	0.00	0.07 to 0.13

M+P-Xylene						

≤ 39 yrs	0.01	0.09	-0.002 to 0.02	0.002	0.9	-0.02 to 0.03

> 39 yrs	0.01	0.03	0.001 to 0.02	0.05	0.00	0.03 to 0.06

O-Xylene						

≤ 39 yrs	0.03	0.12	-0.01 to 0.06	0.01	0.66	-0.05 to 0.07

> 39 yrs	0.02	0.04	0.001 to 0.03	0.11	0.00	0.07 to 0.14

Decane						

≤ 39 yrs	0.01	0.07	-0.001 to 0.02	0.003	0.76	-0.02 to 0.02

> 39 yrs	0.01	0.03	0.001 to 0.01	0.04	0.00	0.03 to 0.05

Tetrachloroethylene				NT	NT	NT

≤ 39 yrs	0.02	0.31	-0.02 to 0.05			

> 39 yrs	0.01	0.002	0.004 to 0.02			

Trichloroethylene						

≤ 39 yrs	0.003	0.84	-0.03 to 0.04	-0.03	0.27	-0.10 to 0.03

> 39 yrs	0.02	0.02	0.003 to 0.03	0.06	0.002	0.03 to 0.10

Toluene	NT	NT	NT			

≤ 39 yrs				-0.004	0.57	-0.02 to 0.01

> 39 yrs				0.02	0.00	0.01 to 0.03

1,4 Dichlorobenzene^§^	0.01	0.39	-0.01 to 0.03			

≤ 39 yrs				-0.03	0.38	-0.10 to 0.04

> 39 yrs				0.11	0.00	0.06 to 0.16

1, 2,4Trimethylebenzene^§^	0.01	0.12	-0.002 to 0.02			

≤ 39 yrs				0.01	0.81	-0.03 to 0.04

> 39 yrs				0.07	0.00	0.05 to 0.09

Undecane^§^	0.003	0.23	-0.002 to 0.01			

≤ 39 yrs				0.01	0.14	-0.004 to 0.03

> 39 yrs				0.03	0.00	0.02 to 0.03

Styrene^§^	0.03	0.36	-0.03 to 0.09			

≤ 39 yrs				-0.07	0.52	-0.29 to 0.15

> 39 yrs				0.34	0.00	0.23 to 0.44

PAHs						

Chrysene^£^				0.10	0.50	-0.19 to 0.38

≤ 39 yrs	0.07	0.57	-0.18 to 0.32			

> 39 yrs	0.20	0.04	0.01 to 0.39			

Fluranthene^£^				0.02	0.68	-0.07 to 0.10

≤ 39 yrs	-0.002	0.96	-0.07 to 0.07			

> 39 yrs	0.09	0.02	0.02 to 0.16			

Phenanthrene^£^				0.01	0.51	-0.02 to 0.05

≤ 39 yrs	0.01	0.67	-0.02 to 0.04			

> 39 yrs	0.04	0.01	0.01 to 0.07			

Pyrene				NT	NT	NT

≤ 39 yrs	0.004	0.92	-0.09 to 0.10			

> 39 yrs	0.08	0.03	0.01 to 0.15			

Anthracene				NT	NT	NT

≤ 39 yrs	0.02	0.88	-0.24 to 0.27			

> 39 yrs	0.25	0.01	0.06 to 0.44			

Benzo(g,h,i)perylene				NT	NT	NT

≤ 39 yrs	-0.01	0.96	-0.33 to 0.31			

> 39 yrs	0.20	0.04	0.01 to 0.39			

Indeno(1,2,3-cd)pyrene				NT	NT	NT

≤ 39 yrs	-0.01	0.98	-0.49 to 0.48			

> 39 yrs	0.34	0.03	0.03 to 0.65			

Benzo(j)fluoranthene				NT	NT	NT

≤ 39 yrs	-0.01	0.98	-0.37 to 0.36			

> 39 yrs	0.26	0.04	0.02 to 0.51			

Dibenz(a,h)anthracene^§^	0.85	0.02	0.13 to 10.57	NT	NT	NT

* Variables in the final models. (i) MNi/1000 BNed models: toxicants (one each time), age (≤ 39/>39 years), toxicant-age interaction, alcohol consumption (regularly/occasionally), vegetable intake (usually/sometimes).

(ii) tail DNA percent models: same as MNi/1000 BNed plus level of education (>12/≤12 years).

^§ ^or ^£ ^Not significant interaction between toxicant and age for comet^§ ^or micronuclei^£ ^assays. Toxicant effect adjusted for the other models variables.

NT: Not Tested because P > 0.1 in univariate association between toxicant and biomarker (table 2).

Notes: Variables entered in the models are those with *P *< 0.1 in univariate analysis. Enter method was used.

Reg. coeff #, regression coefficient number

CI, confidence interval

yrs, years

MNi, micronuclei

BNed; binucleated cells.

### 24 hours urinary 8-oxodG

**Figure 3:**
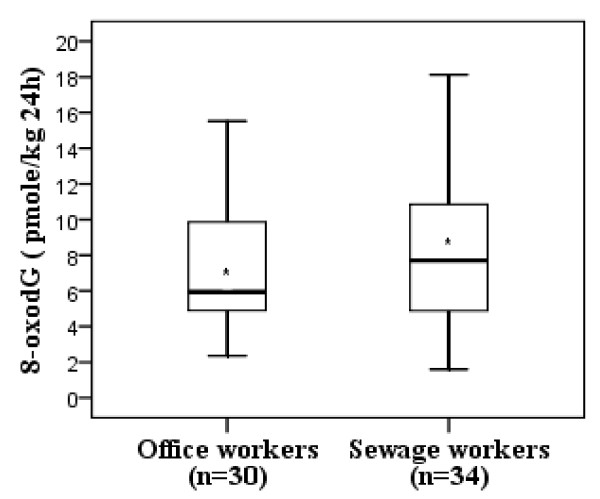
**Box and whisker plot for the 24 hours urinary 8-oxodG level (pmole/kg 24 hours) in sewage and office workers, P = 0.28 for the mean differences**. * Mean 8-oxodG, 8-oxo-2'-deoxyguanosine.

Figure 3 shows the box and whisker plots of 24 hours urinary 8-oxodG in sewage and office workers. There was a slightly (but not significantly) higher mean level in sewage compared to office workers (mean ± SD, 8.26 ± 4.26 pmole/kg 24 h and 7.22 ± 3.32 pmole/kg 24 h respectively, P = 0.28). There was no significant difference in 8-oxodG level regarding other exposure factors mentioned in Table 1. No clear association could be found between the workplace concentrations of the measured pollutants or of total [B(a)P]eq and the level of 24 hours urinary 8-oxodG (data not shown).

## Discussion

This is, to our knowledge, the first work that (i) measured workplace air concentrations of PAHs and VOCs (indicators of external exposure) among underground sewage workers, (ii) applied the HepG2 cells comet and CBMN assays with urine organic extracts, and (iii) assessed the 24 hours urinary 8-oxodG excretion among those workers. The urinary extracts of sewage workers induced significantly greater DNA and chromosomal damage than office workers (Figure 2A and B), suggesting that sewage workers are exposed to substances with genotoxic effects. In contrast to many published monitoring studies that measure specific urinary metabolites, our novel *in vitro *approach measures the overall genotoxicity of urine due to multimedia exposure to complex mixtures of chemicals encountered in the work environment. Those integrated biomarkers reflect exposure from hours to a few days prior to urine sampling. In addition to encompassing the diversity and time variable levels of exposure, it allows to account for their multiple portals of entry into the human body.

In accord with our results, mutagens in the urine of sewage workers with the Ames test have been detected [35]. We found no other reference in the literature that bears on this population. Although results of this study cannot be directly compared with *ex-vivo *assays, other authors have assessed the hazardous potency of this occupational environment. Friis et al. [36] investigated the level of DNA damage on lymphocytes of sewage workers by comet assay and found no difference compared with construction workers. Significant increase in MNi frequency on lymphocytes was reported among asphalt workers [37] while elevated lymphocytes DNA damage by comet assay was shown in workers of a petroleum hydrocarbons facility [38].

In our study, the 24 hours urinary 8-oxodG failed to show a statistically significant difference (probably due to our small sample size) between sewage and office workers, yet average value were slightly greater among the former (Figure 3). Similarly, a study among workers exposed to diesel particles did not show increased levels after a working week [39]. However, several studies demonstrated an increased level of 24 hours urinary 8-oxodG among workers exposed to different sources of genotoxicants like coke oven emissions and ambient air pollutants [27,40].

We attempted to interpret our data in terms of cancer risk based on specific PAHs and benzene workplace air levels that account only for a part of this complex exposure. We found that the Paris city sewage workers experience a substantial lifetime cancer risk via inhalation, ranging from 1.5 × 10^-5 ^to 14.9 × 10^-5^, which is over the acceptable cancer risk ranges defined by American regulatory agencies [33,34,41]. Our results might partially explain the excess in cancer incidence in sewage workers found by other authors [8,9].

The workplace air concentrations of specific pollutants were significantly higher in the sewage than in office workplaces and greater than ambient air concentrations measured by urban background monitors and even in traffic areas (Table 2 and Figure 1). The multivariate analysis revealed significant associations between short term (3 consecutive days) workplace concentrations of several VOCs and both urinary biomarkers, while PAHs were significantly associated with percent DNA tail only (Table 3). These associations were detected among the older but not the younger workers (Table 4). Keeping in mind that these associations should not be causally ascribed to specific compounds, all very much correlated, several hypotheses might be advanced to explain these differences. The two *in vitro *assays have different mechanisms and positive results in comet do not necessarily yield positive ones in CBMN. Indeed, comet assay reflects repairable DNA damage and breaks [42], while CBMN assay reflects chromosomal damage [25]. Noteworthy is that the average VOCs concentrations in this study were all below the French and American recommended occupational permissible exposure limits [43-45]. The observed concentrations for B(a)P are an order of magnitude lower than the provisional limit value proposed by INRS, the occupational security institute in France (150 ng/m^3^, 8-hour TWA). Hence, higher PAHs exposure levels might be needed to elicit a significant observable damage at the chromosomal level on HepG2 cells using CBMN assay [46]. PAHs exposures were also lower than those detected in the workplace air of coke-oven and graphite-electrode producing workers [47]. The studied PAHs and VOCs families differ in their chemical, physical and toxicokinetic properties. Furthermore, they represent only part of the many chemicals to which sewage workers are exposed in the workplace. Clearly, the true exposure in this complex environment is unknown and the possible observed integrated effects could relate to the overall mixture, with synergistic and/or antagonistic interactions [48].

We have no firm explanation for the difference observed across the two age categories. When we analyzed the comet and micronucleus outcomes among older and younger subjects, we found genotoxicity responses on HepG2 cells in both age groups (data available upon request). Table 3, however, shows that these responses significantly differ between the two age groups. Because these results are based on *in vitro *assays [49], this difference cannot be explained by a lower capacity of DNA repair mechanisms in older subjects, nor should it be due to differences in kidney function and excretion rates, as we found no significant difference between younger and older subjects in the 24 hours urinary creatinine or 24 hours urinary volume levels (mean ± SD, 1.2 ± 0.60 g/L and 1.0 ± 0.45 g/L, P = 0.20; and 1.63 ± 0.71 L and 1.60 ± 0.66 L, P = 0.91; respectively). We offer for discussion tentative hypotheses. Firstly, the diversity in tasks of sewage workers according to age might play a role if more experienced subjects are called for activities that incur greater exposure [9]. Contrasts in exposure levels cannot be assessed in our study using the workplace concentrations data stratified according to age, because a work team is usually composed of subjects of all ages and measurements represent exposure levels for all the team members. Differences concerning wearing protections devices are also factors influencing the degree of true individual exposure. Only 32% of sewage workers declared usage of protection equipments (such as rubber gloves, waterproof dressing, rubber boots and goggles), and older ones were less likely to do so than younger ones (P = 0.01). Secondly, many xenobiotics in the workplace (e.g., PAHs and VOCs) are lipophilic, and therefore stored in the fatty tissue [48]. Aged volunteers may have more saturated fatty tissues, a feature that could explain higher release and excretion of such substances in urine [50]. Although *in vitro *assays were performed, another possible route of explanation for the higher comet results among older subjects might implicate cellular aging, assuming that the underlying mechanism is free radical production.

No association was detected between indicators of external exposure and the 24 hours urinary 8-oxodG. One reason might be related to differences in the time dynamics of the two types of measures. While measurements of external exposure represent the last work shift or up to 3 days before, our biomarker of oxidative stress encompasses a much longer period of exposure [12,13]. More important in our view is the fact that the workplace PAHs and VOCs concentrations are poor proxies of the occupational complex exposure that sewage workers experience, so that exposure misclassification may be large. This, in our opinion, gives weight to the integrated exposure approach we propose. Benzene (or any other single PAHs or VOCs we measured) should not be viewed as the sole agents that cause genotoxicity. Rather, we used benzene measurements as a metric for global exposure where benzene is an indicator for an array of other multiple (unmeasured) airborne toxicants that share the same determinants. Similar studies in other complex occupational settings should be conducted to assess the generalizability of our results and how sensitive these urinary biomarkers might be to a variety of mixtures. Once the performance of this approach evaluated, it might be extended to environmental settings such as coastal or soil petroleum pollution and/or contaminated industrial sites.

This study has some limitations. Certain studies had shown that HepG2 cellular line lack several specific enzymes that account for their inability to process some promutagens. In our study, the high formation of MNi in the negative control cultures suggests that HepG2 could also be affected by a genetic instability where they might unpredictably acquire a mutated phenotype. On the other side, many studies had reported that this cellular line retained certain activities of various phase I and phase II enzymes which play a key role in the activation and detoxification of various promutagens. They, therefore, could be a good in vitro model to study genotoxicity and DNA damage [51,52]. To date, however, human hepatocytes cells are the preferred and the most promising alternative cellular line due to their high metabolic activity for biotransformation similar to human liver [53]. They express phase I enzymes at significantly higher levels than HepG2 cells. However, they are currently available at a cost almost prohibitive, there is a shortage of available human liver material and they pose the problem of inter-individual variations. Additionally, primary hepatocytes do not proliferate and lose their metabolic activity after some weeks. The small sample size and the possibility of lack of adjustment for unknown/unconcontrolled confounders as well as some biases like recall (questionnaires reporting) or healthy worker effect (mainly among sewage workers) could also be considered as one of the study limitations. A larger sample size would have yielded more consistent results and conclusions but lack of power is not an explanation. To check this, we made a post hoc power assessment, considering that urines from an unexposed population are not mutagenic in theory, so that both genotoxicity tests should be negative. Thus, assuming a 1% prevalence of genotoxic response among office workers, our sample size (30 and 34 subjects in the two groups) is sufficient to highlight a prevalence of anomalies of nearly 25% in the exposed group. For urinary 8-oxodG, the expected standard deviation value is 10.8. With our sample size, we could detect nearly 33% modification of its mean value. These estimates were based on a type І error (α) of 5% and a power expectation of 80%.

## Conclusions

Sewage workers are exposed through different pathways to a variety of toxicants. We propose an integrated approach to assess exposure to a blend of genotoxicants using noninvasive urinary biomarkers. Nevertheless, lack of adjustment for unknown confounders cannot be ruled out in our small study population. Also, because of the multi-factorial nature in the production of 8-oxodG - a fraction of the repair metabolites of 8-oxo-guanine, the increased urinary excretion of 8-oxodG should be linked to cancer risk with great caution. However appealing the simplicity of the approach may be, these results warrant verification in other studies and exposure settings.

## List of abbreviations used

8-oxodG: 8-oxo-2'-deoxyguanosine; B(a)P: Benzo(a)pyrene; [B(a)P]eq: Benzo(a)pyrene equivalent concentration; BNed: Binucleated cells; CBMN: Cytokinesis block micronucleus assay; MNi: Micronuclei; NDI: Nuclear division index; PAHs: Polycyclic aromatic hydrocarbons; TEFs: Toxicity equivalent factors; TWA: Time weighted average; U.S. EPA: United States Environmental Protection Agency; VOCs: Volatile organic compounds

## Competing interests

The authors declare that they have no competing interests.

## Authors' contributions

HA drafted the manuscript, performed the genotoxicity assays and the statistical analysis. LF, DZN, CP and ISM participated in the design and coordination of the study protocol, statistical analysis and in drafting the manuscript. Other authors reviewed the manuscript, provided further contributions and suggestions. All authors read and approved the final manuscript.
